# Measurement Variability of Persistent Pulmonary Subsolid Nodules on Same-Day Repeat CT: What Is the Threshold to Determine True Nodule Growth during Follow-Up?

**DOI:** 10.1371/journal.pone.0148853

**Published:** 2016-02-09

**Authors:** Hyungjin Kim, Chang Min Park, Yong Sub Song, Leonard Sunwoo, Ye Ra Choi, Jung Im Kim, Jae Hyun Kim, Jae Seok Bae, Jong Hyuk Lee, Jin Mo Goo

**Affiliations:** 1 Department of Radiology, Seoul National University College of Medicine, Seoul, Korea; 2 Aerospace Medical Group, Air Force Education and Training Command, Jinju, Korea; 3 Institute of Radiation Medicine, Seoul National University Medical Research Center, Seoul, Korea; 4 Cancer Research Institute, Seoul National University, Seoul, Korea; 5 Department of Radiology, SMG-SNU Boramae Medical Center, Seoul, Korea; 6 Department of Radiology, Kyung Hee University Hospital at Gangdong, Seoul, Korea; Peking University People Hospital, CHINA

## Abstract

**Purpose:**

To assess the measurement variability of subsolid nodules (SSNs) in follow-up situations and to compare the degree of variability between measurement metrics.

**Methods:**

Two same-day repeat-CT scans of 69 patients (24 men and 45 women) with 69 SSNs were randomly assigned as initial or follow-up scans and were read by the same (situation 1) or different readers (situation 2). SSN size and solid portion size were measured in both situations. Measurement variability was calculated and coefficients of variation were used for comparisons.

**Results:**

Measurement variability for the longest and average diameter of SSNs was ±1.3 mm (±13.0%) and ±1.3 mm (±14.4%) in situation 1, and ±2.2 mm (±21.0%) and ±2.1 mm (±21.3%) in situation 2, respectively. For solid portion, measurement variability on lung and mediastinal windows was ±1.2 mm (±27.1%) and ±0.8 mm (±24.0%) in situation 1, and ±3.7 mm (±61.0%) and ±1.5 mm (±47.3%) in situation 2, respectively. There were no significant differences in the degree of variability between the longest and average diameters and between the lung and mediastinal window settings (p>0.05). However, measurement variability significantly increased when the follow-up and initial CT readers were different (p<0.001).

**Conclusions:**

A cutoff of ±2.2 mm can be reliably used to determine true nodule growth on follow-up CT. Solid portion measurements were not reliable in evaluating SSNs’ change when readers of initial and follow-up CT were different.

## Introduction

During the past decade, there has been growing awareness of the significance of pulmonary subsolid nodules (SSNs), also known as ground-glass nodules (GGNs), given that the probability of malignancy is higher in SSNs than in solid nodules [1] and that SSNs reflect the histologic spectrum of pulmonary adenocarcinoma [2]. However, as SSNs are known to include various histologic backgrounds including many benign conditions [3], differentiation between benign and malignant SSNs is of utmost importance for the proper diagnosis of lung cancer and to avoid unnecessary invasive diagnostic procedures.

Invasive adenocarcinomas appearing as SSNs are typically recognized through a nodule size >10 mm or the presence of internal solid portions >5 mm at any single time point [4–6]. In follow-up situations, an increase in size, attenuation or new appearance of solid portions within the nodule can potentially indicate malignant growth [7]. Therefore, determination of nodule growth is key to determining the management strategy during follow-up. However, measurement variability, which would be the threshold to determine true nodule growth, of small SSNs has not yet been established.

With respect to the analysis of measurement variability, replication of the exact parameters of actual clinical practice is an important element. In routine practice, radiologists measure nodule size on CT side-by-side with the previous CT for comparison along with all prior measurement data assessed by either the same or different readers. Follow-up measurements are not performed on a single CT without information of the prior exam in real practice. Nevertheless, this aspect was not reflected in the previous measurement variability studies of SSNs to date [7–12]. Measurements in previous studies were performed independently between readers without reference on prior CT images.

Therefore, in the present study, we aimed to simulate a situation in which the same reader (situation 1) and different readers (situation 2) performed follow-up CT measurements, with the initial CT scans placed side-by-side along with prior measurement data, using two same-day repeat CT scans. First, we assessed the measurement variability range of whole nodules and solid portion size in each situation. Second, we compared the degree of measurement variability between the longest and average diameters for whole nodule size and between the lung window and mediastinal settings for solid portion size.

## Materials and Methods

This retrospective analysis was approved by the institutional review board of Seoul National University Hospital and written informed consent was waived (IRB No. 1310-107-530) as the data were analyzed retrospectively and anonymously.

### Patients

Our study population was comprised of 73 patients who were included in a previously published prospective study [7], which investigated semi-automated volumetric measurement variability of SSNs. In the original study, patients with persistent SSNs (>5 mm and <20 mm; solid portion ≤5 mm on mediastinal window setting for part-solid GGNs) who underwent two same-day repeat CT scans under the informed consent from November 2011 to June 2012 were enrolled. The inclusion criteria targeted SSNs that required annual surveillance CT for a minimum of 3 years as recommended by the Fleischner Society [13].

Among the 73 patients, four patients were excluded due to loss of available thin-section CT data. As a result, 69 patients were finally included for analysis (24 men and 45 women; age, 62.21±9.74 years for men and 53.93±12.05 years for women). In patients with multiple nodules, a single SSN was selected for the analysis in the present study. As nodule and solid portion size determine the management strategy, the largest lesion was chosen for patients with multiple pure GGNs. For the patients with both pure and part-solid GGNs, part-solid GGNs were chosen. Therefore, 69 patients with 69 SSNs were analyzed. The demographic detail and radiation dosage are as described in our previous study [7].

### CT Acquisition

Two consecutive unenhanced CT examinations were performed within 10 minutes, and every patient was instructed to hold their breath in full inspiration during each CT scan. All CTs were taken using Somatom Sensation-16 (Siemens Medical Solutions, Forchheim, Germany). Scanning parameters were as follows; detector collimation, 0.75 mm; beam pitch, 1.0; reconstruction increment, 1.0 mm; slice thickness, 1.0 mm; rotation time, 0.5 second; tube voltage, 120 kVp; tube current, 60 mAseff; and matrix, 512 × 512. Images were reconstructed using a medium sharp reconstruction algorithm. After the first CT examination covering the entire thorax, patients were asked to leave the CT scanner, and then were asked to come back to the table, and a second limited range scan with a new localizer scanogram covering only SSNs to reduce additional radiation exposure was performed within 10 minutes.

### Nodule Classification and Size Measurement

Image analyses and measurements were performed in two distinct situations. First, two same-day repeat-CT scans were randomly assigned as initial or follow-up scans. In situation 1 or *same reader follow-up*, the initial and follow-up CT scans were both read by the same reader. In situation 2 or *replaced reader follow-up*, the reader was changed for the follow-up CT measurement.

In both situations, measurements were performed with electronic caliper and caliper marks (annotations) were neither saved nor provided to the readers for the follow-up measurements. Measurement data were recorded on a separate Excel file and were collected and redistributed by one author (H.K.) who did not participate in the image analysis. For solid portion measurement, readers were instructed that the measurement orientation or image slice on which to measure the solid portion could be different between window settings in a single CT scan as the largest solid portion may appear at different image slice between window settings according to the attenuation.

All readers were blinded from the purpose of the study and from knowing how much time had passed between the two scans. Patient identifiers and study dates were removed from the image headers. The order of reading was randomized by patient. For analysis, adjusting the window width and level of images and usage of zoom function of picture archiving and communication system were available to the readers as necessary. Image display layout on a monitor was chosen according to the reader’s preference.

#### Situation 1

**Fig 1:**
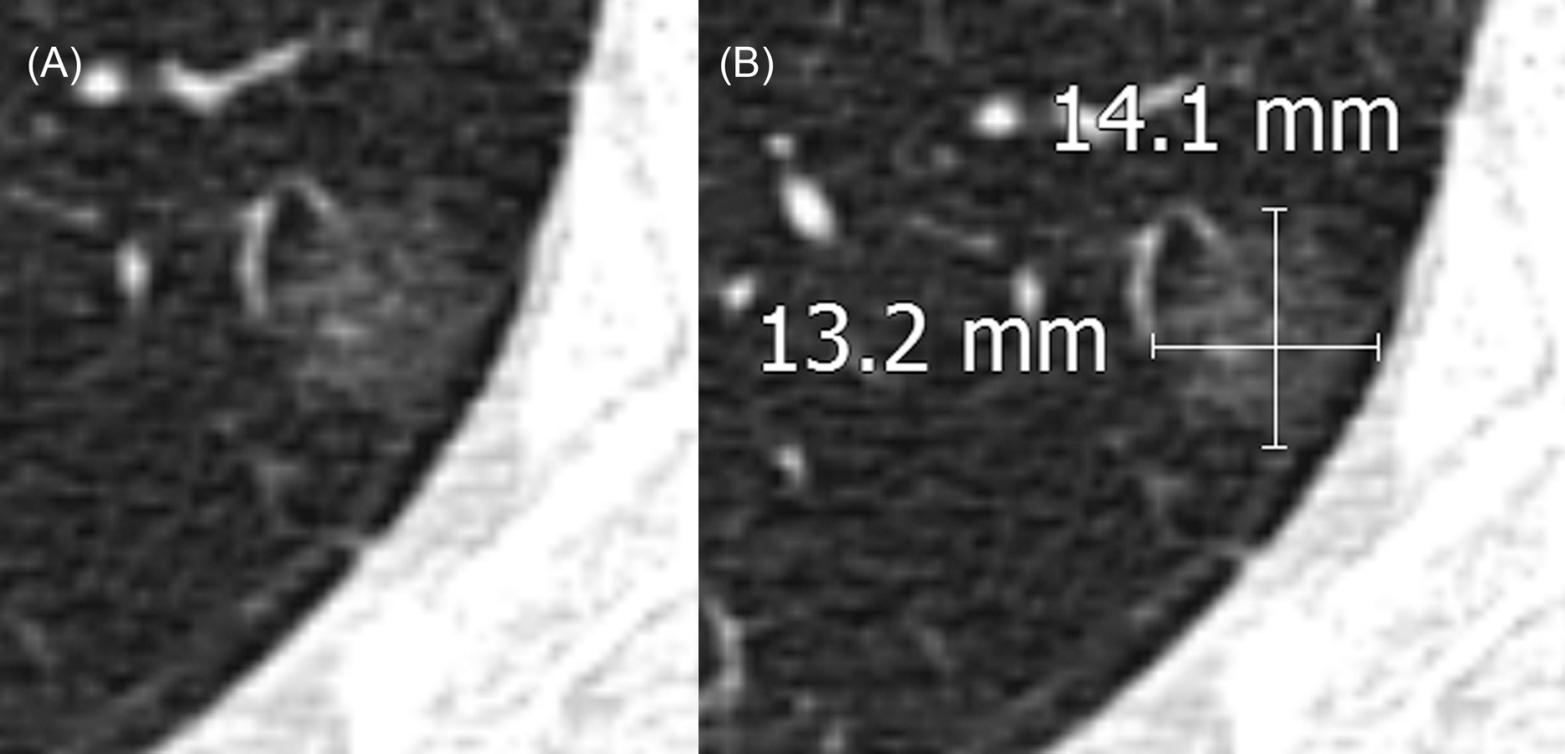
A pure ground-glass nodule in the right upper lobe of the lung in a 73 year-old female. (A) The subsolid nodule has a less distinct margin than the solid nodule. (B) The longest diameter along with its maximum perpendicular diameter was measured. Longest diameter measurement differences between scans ranged from -1.8 to 1.3 mm when the follow-up CT reader was different from that at initial CT.

**Fig 2:**
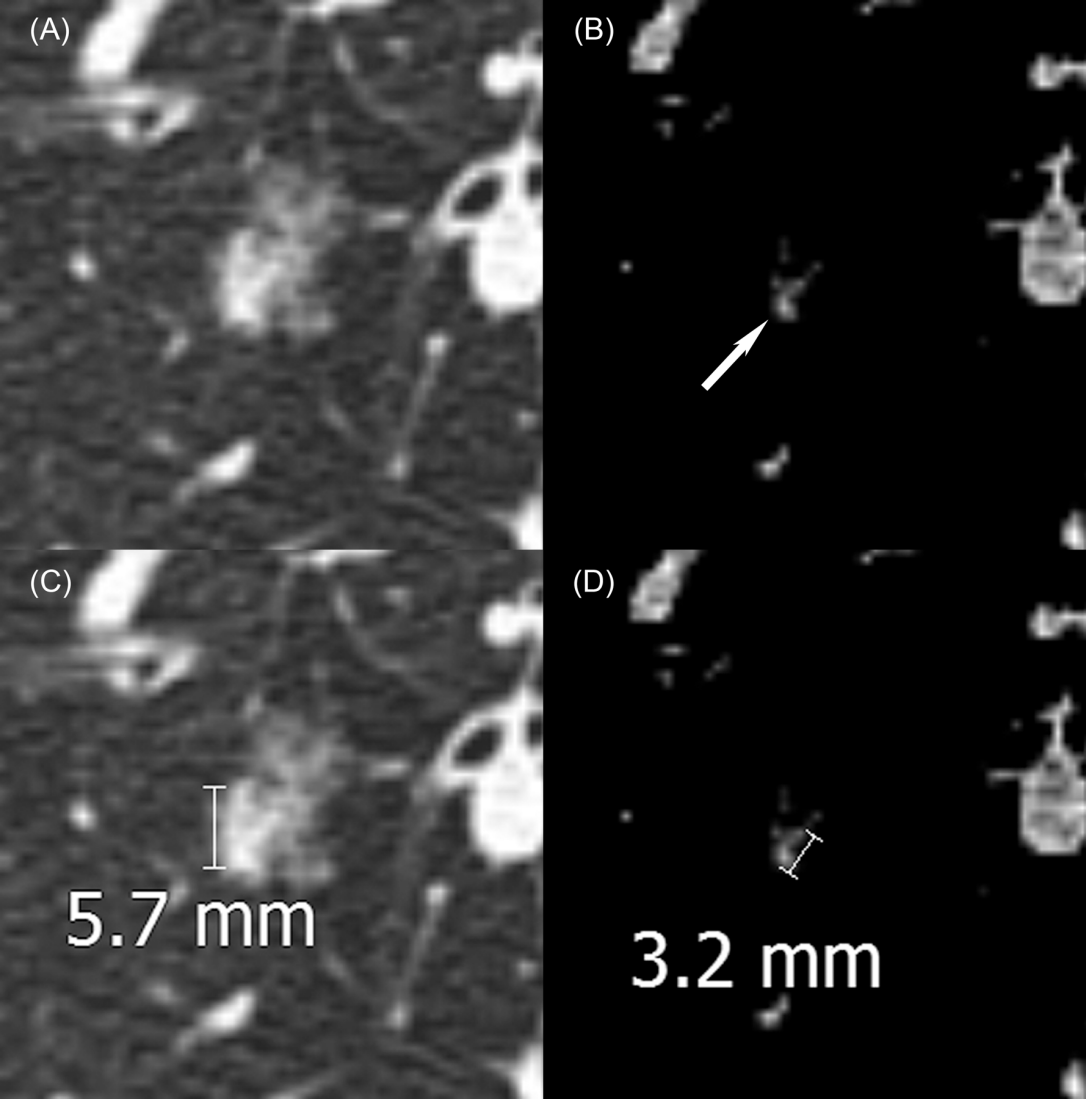
A 55 year-old female with a part-solid nodule in the right lower lobe of the lung. The internal solid portion was identified on both (A) lung window and (B) mediastinal window settings (arrow). Measurement differences between scans on replaced reader follow-up ranged (C) from -3.3 to 0.2 mm on lung window and (D) from -2.0 to 0.5 mm on mediastinal window.

CT images were viewed by four radiologists (Y.S.S., L.S., Y.R.C. and J.I.K. with 4, 4, 3 and 11 years of experience in CT, respectively). These radiologists were not involved in the previous prospective study [7]. Radiologists were first asked to classify whether the nodules were pure or part-solid GGNs according to the definition suggested by the Fleischner Society [13]. Thereafter, the longest diameter and its maximal perpendicular diameter were measured for each nodule on lung window setting using an electronic caliper (Fig 1). The averaged uni-dimensional diameter was calculated. Subsequently, the solid portion size (longest diameter) was measured for part-solid GGNs on both lung and mediastinal window settings, that is, the solid portion was measured twice for each lesion (Fig 2). Lung window setting was adopted since the small solid portion can be better visualized with greater sensitivity on the lung window than on the mediastinal window [14]. Mediastinal window setting was also chosen as it is supposed to offer higher reproducibility of solid portion measurement and it is recommended by the Fleischner society guideline [13, 15].

After the initial nodule classification (pure or part-solid GGNs) and measurements were made, the radiologists viewed the second scan, which was assigned as the follow-up CT, side-by-side along with the initial CT as well as the measurement data they had made. Therefore, the same reader conducted the follow-up CT measurement in this situation in the same fashion as the initial scan. All reading sessions were separated by an interval of 4 weeks.

#### Situation 2

In situation 2, four different radiologists (three residents, J.H.K., J.S.B., J.H.L. with 3 years of experience in CT; and a senior attending radiologist, J.M.G. with 25 years of experience in CT) were given a set of nodule classification and size data described to them as the ‘initial CT measurement’. This dataset was randomly selected from the situation 1 pooled data. In addition, two sets of CT scans randomly assigned as initial and follow-up CT scans were provided and the readers were asked to perform the nodule classification and measurements in the same manner as in situation 1 on the ‘follow-up CT’ side-by-side along with the initial CT and measurement data obtained from situation 1. Finally, the readers were requested to determine nodule growth on the follow-up scan subjectively (stable, growth, or shrinkage).

### Statistical Analysis

With respect to the analysis of nodule classification among readers, overall percentage agreement and multirater Fleiss ĸ statistics were calculated for the eight classification results per nodule by the situation 1 readers. Then, the percentage of consistent nodule classifications between the two scans in situation 1 (intra-reader nodule classification consistency) and in situation 2 (inter-reader nodule classification consistency) was obtained. Nodule classification consistency was compared between the two situations using the McNemar test. Cohen ĸ statistics were also used to measure each reader’s classification agreement between scans.

In terms of measurement variability, measurement differences between the two scans were calculated in millimeters and in percentages for each metric. Thereafter, randomized distribution of the calculated differences was repeated 1000 times [16]. 95% limits of agreement for measurement differences, which represent the range of variability, were calculated as the mean ±2 standard deviations (SDs) [16]. Analysis of measurement variability was performed for the whole nodule (longest and average diameter) and solid portion size (longest diameter on lung window and mediastinal window settings), respectively.

To compare the degree of measurement variability between the same and replaced reader follow-up readings, as well as between the longest and average diameter of whole nodule size and between lung and mediastinal window settings for solid portion size, we calculated coefficients of variation (CVs) [8]. CV was calculated as the SD divided by the mean [8]. Paired Wilcoxon test or Mann-Whitney U test was performed for comparisons, as appropriate, after the Kolmogorov-Smirnov’s test for variables.

For subgroup analysis, CVs of whole nodule size were compared between pure and part-solid GGNs and between nodules <1 cm and ≥1 cm. Nodule type was determined by majority rule among the eight classifications per nodule obtained in situation 1. Nodule size was estimated from the average of eight measurements per nodule in situation 1.

With the subjective nodule growth assessment data acquired in situation 2, the misinterpretation rate (percentage of nodule growth or shrinkage) was evaluated as there was actually no change between the two scans. The misinterpretation rate was compared between the senior attending radiologist (J.M.G) and residents (J.H.K., J.S.B. and J.H.L.) using Fisher’s exact test.

All statistical analyses were performed using R software, version 3.2.2 (http://www.R-project.org) and SPSS 19.0 (IBM SPSS Statistics, Armonk, NY). A P value of less than 0.05 was considered to indicate a significant difference. Bonferroni correction was applied for multiple comparisons. All data of nodule classification and measurements are available in S1 Dataset.

## Results

### Nodule Classification Agreement

Among the 69 SSNs, 53 were pure and 16 were part-solid GGNs. Overall percentage nodule classification agreement in situation 1 was 91.67±13.84% and multirater Fleiss ĸ value was 0.66 (95% confidence interval [CI]: 0.62, 0.71). Intra-reader nodule classification consistency (situation 1) was 97.83% (270/276) and the ĸ values of each reader were 0.96 (95% CI: 0.88, 1.00), 1.00 (95% CI: 1.00, 1.00), 0.84 (95% CI: 0.71, 0.98) and 1.00 (95% CI: 1.00, 1.00). Inter-reader nodule classification consistency (situation 2) was 95.29% (263/276) (p = 0.167) and the ĸ values of each reader were 0.83 (95% CI: 0.67, 0.99), 0.80 (95% CI: 0.63, 0.97), 0.96 (95% CI: 0.88, 1.00) and 0.88 (95% CI: 0.75, 1.00).

### Whole Nodule Size Measurement Variability

For whole nodule size measurement, mean longest diameter was 11.0±3.3 mm (range, 4.7–22.3 mm) based on situation 1 measurements. SD of measurement differences was 0.7 mm (6.5%) in situation 1 and 1.1 mm (10.5%) in situation 2, indicating that the range of measurement variability was ±1.3 mm (±13.0%) and ±2.2 mm (±21.0%), respectively.

Mean average diameter was 10.0±2.9 mm (range, 4.4–21.4 mm) based on situation 1 measurements. SD of measurement differences was 0.7 mm (7.2%) in situation 1 and 1.0 mm (10.6%) in situation 2, indicating that the range of measurement variability was ±1.3 mm (±14.4%) and ±2.1 mm (±21.3%), respectively. A detailed summary of this data can be found in Table 1.

**Table 1:** Measurement variability of whole nodules and solid portion size on follow-up CT scans.

		Standard deviation (mm)	Range of measurement variability (mm)	Standard deviation (%)	Range of measurement variability (%)
Whole nodule size	Longest diameter (S1)	0.7	±1.3	6.5	±13.0
	Longest diameter (S2)	1.1	±2.2	10.5	±21.0
	Average diameter (S1)	0.7	±1.3	7.2	±14.4
	Average diameter (S2)	1.0	±2.1	10.6	±21.3
Solid portion size	Lung window (S1)	0.6	±1.2	13.5	±27.1
	Lung window (S2)	1.8	±3.7	30.5	±61.0
	Mediastinal window (S1)	0.4	±0.8	12.0	±24.0
	Mediastinal window (S2)	0.8	±1.5	23.6	±47.3

S1, situation 1 (same reader follow-up); S2, situation 2 (replaced reader follow-up)

### Solid Portion Size Measurement Variability

Mean solid portion size was assessed separately in each situation as the nodule classification was slightly different between situations. Mean solid portion size on the lung window setting was 5.4±2.8 mm (range, 1.6–15.0 mm) in situation 1 and 5.8±2.8 mm (range, 1.7–13.5 mm) in situation 2. SD of measurement differences was 0.6 mm (13.5%) in situation 1 and 1.8 mm (30.5%) in situation 2, indicating that the range of measurement variability was ±1.2 mm (±27.1%) and ±3.7 mm (±61.0%), respectively.

Mean solid portion size on the mediastinal window setting was 3.3±2.1 mm (range, 0–8.8 mm) in situation 1 and 3.7±2.1 mm (range, 0–8.6 mm) in situation 2. Non-visible lesions in the mediastinal window were not included for the calculation of measurement variability. SD of measurement differences was 0.4 mm (12.0%) in situation 1 and 0.8 mm (23.6%) in situation 2, indicating that the range of measurement variability was ±0.8 mm (±24.0%) and ±1.5 mm (±47.3%), respectively. The data are summarized in Table 1.

### Comparison of Measurement Variability

CVs of all metrics were significantly higher in replaced reader follow-up than in same reader follow-up (all p<0.001), indicating that the measurement variability was larger when the reader was changed for follow-up CT measurement. There were no significant differences between CVs of the longest and average diameters for whole nodule size and between CVs of solid portion size on lung and mediastinal settings in both situations (p>0.05). The mean CV of each measurement and the P value of each comparison are summarized in Tables 2 and 3.

**Table 2:** Coefficient of variation (CV) comparisons of whole nodule size measurements.

Whole nodule size measurement	Mean CV		P value
	Same reader follow-up	Replaced reader follow-up	
**Longest diameter**	0.036	0.057	<0.001
**Average diameter**	0.036	0.057	<0.001
**P value**	0.083	0.558	

**Table 3:** Coefficient of variation (CV) comparisons of solid portion size measurements.

Solid portion size measurement	Mean CV		P value
	Same reader follow-up	Replaced reader follow-up	
**Longest diameter on lung window**	0.067	0.131	<0.001
**Longest diameter on mediastinal window**	0.064	0.126	<0.001
**P value**	0.844	0.208	

CVs of the longest and average diameters were significantly higher in nodules <1 cm than in nodules ≥1 cm in situation 1 (all p<0.001). The CV of the longest diameter was also significantly higher in nodules <1 cm in situation 2 (p<0.001), however, there were no significant differences in CV in average diameter between the two size groups in situation 2 (p = 0.077). The CV of the longest diameter was larger than that of the average diameter for nodules <1 cm in both situations (p<0.001). Pure GGNs showed a larger CV than part-solid GGNs in situation 1 for both metrics (p<0.001), however, such statistical significance was not found in situation 2 after the significance level was reduced to an α–adjusted P level of 0.0125. Detailed data are displayed in Tables 4 and 5.

**Table 4:** Subgroup analysis according to nodule size and type in situation 1 (same reader follow-up).

	Mean CV			Mean CV		
	<1 cm	≥1 cm	P value	Pure GGN	Part-solid GGN	P value
**Longest diameter**	0.044	0.030	<0.001	0.038	0.027	<0.001
**Average diameter**	0.037	0.028	<0.001	0.037	0.036	<0.001
**P value**	<0.001	0.018		0.242	0.429	

CV, coefficient of variation; GGN, ground-glass nodule

**Table 5:** Subgroup analysis according to nodule size and type in situation 2 (replaced reader follow-up).

	Mean CV			Mean CV		
	<1 cm	≥1 cm	P value	Pure GGN	Part-solid GGN	P value
**Longest diameter**	0.072	0.049	<0.001	0.058	0.058	0.145
**Average diameter**	0.055	0.053	0.077	0.061	0.052	0.022
**P value**	<0.001	0.538		0.200	0.969	

CV, coefficient of variation; GGN, ground-glass nodule

### Growth Misinterpretation Rate

The overall misinterpretation rate of nodule growth (growth or shrinkage) in situation 2 was 10.87% (30/276; 20 cases in pure GGNs and 10 cases in part-solid nodules). All 30 misinterpretation cases were from residents and none of the cases were interpreted as growth or shrinkage by the senior attending radiologist (p<0.001).

## Discussion

In this study, we demonstrated that the maximum measurement variability was ±2.2 mm (±13.0%) for the longest diameter of whole nodules, with no significant differences in the degree of variability between the longest and average diameters. As for measurement of the solid portion, the maximum measurement variability was ±3.7 mm (±61.0%), which implies that solid portion measurements are unreliable on the lung window setting when the reader is changed at follow-up CT. Reading using the mediastinal window led to smaller absolute variability (±1.5 mm; ±47.3%), however, CV comparisons showed no statistical significances between the two settings. In addition, measurement variability was shown to significantly increase when the reader of follow-up CT was changed from that at initial CT.

Evaluating the measurement variability of SSNs is a clinically important issue in that the determination of true growth on follow-up CT based on a certain threshold would guide future management in diametrically opposed ways of whether to undergo serial surveillance or to be biopsied or surgically resected. To date, a vast number of studies have focused on analyzing measurement variability [7–11], however, the measurements were performed independently between readers without reference on prior exams, which is far from reality. Our present study is the first to reveal the inter-reader measurement variability in the follow-up setting with side-by-side reading of the initial scans along with prior measurement data open to the follow-up reader. The two previous studies on solid nodules with a similar study design performed sequential measurements with the same reader and calculated the measurement differences [16, 17]. Therefore, true inter-reader variability was not reflected in those studies.

For the longest diameter measurement variability, de Hoop et al. [8] reported inter-reader measurement variability as -2.8 to 3.3 mm in their analysis of 52 SSNs (mean, 13.9 mm). In another study, Kakinuma et al. [9] reported the variability range as -3.1 to 2.5 mm in their analysis of 10 SSNs (mean, 10.4 mm). In addition, Kim et al. [10] demonstrated that the inter-reader variability was -3.3 to 1.8 mm using model-based iterative reconstruction with 47 SSNs (mean, 11.8 mm). In our study, the variability measured to be ±2.2 mm in the replaced reader follow-up, which is smaller than that reported previously in the literature. There would be two main reasons for the discrepancy. First, side-by-side reading with initial CT measurement data ‘open’ to the follow-up readers might have reduced the variability. Second, the repeated random distribution step and multiple reader data probably decreased the random error. Our study results correspond with the measurement variability suggested in the recently published British Thoracic Society guidelines [18] and support the use of ‘2 mm’ threshold in determining true nodule growth.

With regard to the solid portion, it can be easily expected that the variability for measuring lesions that are ≤5 mm would be substantial. Indeed, in our study, the maximum measurement variability was ±3.7 mm at replaced reader follow-up using the lung window setting, which was comparable to the results reported by Hwang et al. [19] (−3.6 to 3.0 mm) analyzing 197 part-solid nodules (mean solid portion size, 1.1±0.7 cm). Variability on the mediastinal window was observed to be smaller (±1.5 mm), however, this was still approximately 40% of the mean solid portion size. Large variation on the lung window might be due to the difficulty in delineating the margin of solid portion from the ground-glass background, that is, the contrast resolution is relatively lower on the lung window than on the mediastinal window. On the other hand, the decreased solid portion size and/or adjacent vessels can be the probable causes of high variability on the mediastinal window.

We do not believe at this time that reproducible measurements of solid portions can be feasible when the reader of initial and that of follow-up CTs are different. Subjective judgment of an increase in the solid portion may not be trustworthy solely with size comparisons considering such large measurement variability, although growth assessment in real practice would also be affected by the qualitative evaluation of the anatomical relationship between the solid portion and surrounding background. Using the standardized image analysis protocol with fixed image display settings including window width/level and image layout among the readers and saving the electronic caliper annotations on the images to replicate the prior measurement at follow-ups may help reduce the measurement variability. One possible alternative, however, would be computer-aided volumetry [20]. Indeed, semi-automated three-dimensional diameter or effective diameter measurement with volume or mass estimation may reduce measurement variability [7, 12, 21–23] and enable more sensitive detection of the growth of the solid portion. Scholten et al. [22] reported that the semi-automatic measurements of effective diameter for SSNs were significantly smaller than the manual diameter measurements and that the semi-automatic measurements of diameter, volume and mass showed good inter-observer and inter-scan agreement [12, 22].

In terms of CV comparisons, we observed no significant differences between the longest and average diameters of whole nodule size. However, given that the measurement variability for lesions <1 cm was smaller for the average diameter, we suggest that the follow-up of small SSNs be gauged using the average diameter. As for the solid portion, both the absolute and relative measurement variability was observed to be considerably smaller on the mediastinal window than on the lung window, although CV comparisons showed no statistical significance.

One of our final interesting findings was that the overall nodule growth misinterpretation rate was shown to be 10.87%. We can assume that the measurement variability most likely may have confused the readers of situation 2 in the evaluation of nodule growth. Nevertheless, one experienced reader was able to correctly classify all nodules as ‘stable’ in all cases. Therefore, we presume that the empirical knowledge regarding the measurement variability and the comparison of structural interrelations of the experienced reader may have helped produce the right decision. Accordingly, we believe that recognition of the range of variability of SSNs in clinical practice may help radiologists determine nodule growth more reliably.

We acknowledge a number of limitations to this study. First, measurement variability factors related to CT scanning were not involved in this study. As the patients underwent CT scans on a single scanner with fixed parameters, the influence of factors such as CT scanners, radiation doses, slice thicknesses or reconstruction algorithms on measurement variability was not considered. Second, the number of part-solid GGNs included was small and the range of the solid portion size was limited. Third, solid portion measurements were performed using the longest diameter although the Fleischner Society recommends the average diameter for the mensuration of both the whole nodule and solid portion size [13]. However, the solid portion involved in the present study was generally small (≤5 mm) and it was not practical to obtain both the longest and average diameter in those cases.

In conclusion, a cutoff of ±2.2 mm (±13.0%) for whole nodule size can be reliably used to determine true nodule growth on follow-up CT of SSNs. Solid portion measurements were not reliable in evaluating SSNs’ change when readers of initial and follow-up CT were different.

## Supporting Information

S1 Dataset Nodule classification results and measurement data. (XLSX)
